# Identification and Validation of a Stable Major-Effect Quantitative Trait Locus for Kernel Number per Spike on Chromosome 2D in Wheat (*Triticum aestivum* L.)

**DOI:** 10.3390/ijms241814289

**Published:** 2023-09-19

**Authors:** Zhi Li, Qinyi Luo, Yawen Deng, Ke Du, Xinli Li, Tianheng Ren

**Affiliations:** 1 State Key Laboratory of Crop Gene Exploration and Utilization in Southwest China, Sichuan Agricultural University, Chengdu 611130, China; lizhi@sicauu.edu.cn; 2 College of Agronomy, Sichuan Agricultural University, Chengdu 611130, China; qinyiluo333@163.com (Q.L.); 202000379@stu.sicau.edu.cn (Y.D.); dk18381089612@163.com (K.D.); 18728170209@163.com (X.L.); 3 Key Laboratory of Plant Genetics and Breeding at Sichuan Agricultural University of Sichuan Province, Chengdu 611130, China

**Keywords:** wheat, QTL mapping, KNPS, KASP, yield

## Abstract

A recombinant inbred line population including 371 lines was developed by a high kernel number per spike (KNPS) genotype T1208 and a low KNPS genotype Chuannong18 (CN18). A genetic linkage map consisting of 11,583 markers was constructed by the Wheat55K SNP Array. The quantitative trait loci (QTLs) related to KNPS were detected in three years. Eight, twenty-seven, and four QTLs were identified using the ICIM-BIP, ICIM-MET, and ICIM-EPI methods, respectively. One QTL, *QKnps.sau-2D.1*, which was mapped on chromosome 2D, can explain 18.10% of the phenotypic variation (PVE) on average and be considered a major and stable QTL for KNPS. This QTL was located in a 0.89 Mb interval on chromosome 2D and flanked by the markers *AX-109283238* and *AX-111606890*. Moreover, *KASP-AX-111462389*, a Kompetitive Allele-Specific PCR (KASP) marker which closely linked to *QKnps.sau-2D.1*, was designed. The genetic effect of *QKnps.sau-2D.1* on KNPS was successfully confirmed in two RIL populations. The results also showed that the significant increase of KNPS and 1000-kernel weight (TKW) was caused by *QKnps.sau-2D.1* overcoming the disadvantage due to the decrease of spike number (SN) and finally lead to a significant increase of grain yield. In addition, within the interval in which *QKnps.sau-2D.1* is located in Chinese Spring reference genomes, only fifteen genes were found, and two genes that might associate with KNPS were identified. *QKnps.sau-2D.1* may provide a new resource for the high-yield breeding of wheat in the future.

## 1. Introduction

Wheat is one of the most important food crops in the world. The annual cultivated area of wheat is approximately 220 million hectares, and the annual output of wheat is more than 600 million tons (http://apps.fas.usda.gov/psdonline/app/index.html#/app/downloads, accessed on 31 December 2022). Wheat is the staple food of 30% of the population of the world, and with the population of the world expected to reach nine billion by the 2050s, wheat production will need to increase by 60% to meet the demand [1,2]. China is one of the largest wheat producers, with a sown area of about 23 million hectares, and the output of wheat is about 135 million tons (https://www.gov.cn/lianbo/bumen/202307/content_6892112.htm, accessed on 31 December 2022). Sichuan Province is one of the main wheat-producing areas in China and is a typical rain-fed plus irrigated planting area. Due to the short sunshine and weak light in this region, the wheat yield and quality are significantly lower than that in the wheat-growing regions in northern China [3]. The average wheat yield in Sichuan was only about 12 tons per hectare in 2023. 

In the face of an increasing population and decreasing arable land area, increasing wheat yield is an important way to prevent famine in the world. The spike number per square meter (SN), 1000-kernel weight (TKW), and kernel number per spike (KNPS) are the three most important factors for wheat yield [4,5]. Many studies have shown that the SN, TKW, and KNPS of wheat have continued to increase in recent years, which has played an important role in the improvement of wheat yield [6,7,8]. However, these three factors are mutually restricted. An increase in KNPS often leads to a decrease in SN and TKW [9].

Among the yield components, KNPS has the greatest effect on yield, and its potential to increase yield is also the greatest [10,11,12,13]. The improvement of KNPS is the key goal of wheat breeding for a high yield. However, kernel-related traits are controlled by multiple genes and susceptible to environmental influences. Therefore, exploring major and stable QTLs and developing cost-effective molecular markers can reduce input and improve breeding efficiency, which is of great significance for wheat yield improvement. To date, there have been several reports of QTLs that can improve KNPS in wheat and distribute on 1A, 2A, 4A, 6A, 7A, 2B, 4B, 5B, 7B, 6D, and 7D chromosomes [14,15,16,17,18]. However, most of the reported QTLs were positive for KNPS and were also negative for TKW [4,19,20]. Many genes related to KNPS were cloned, such as *GN1a* [21], *DEP1* [22], *TAW1* [23], *FON4* [24], etc. However, most of them were cloned from rice. Much fewer genes related to KNPS have been cloned in wheat, such as *TaTEF-7A* and *GNI-A1* [5,25]. *GNI-A1* was cloned from the QTL for KNPS on chromosome 2AL and encoded HD-ZIP type I transcription factor. With the mutation with *GNI-A1*, wheat was found to be able to produce more KNPS and a significant effect on kernel size was not observed [5]. Moreover, there are currently too few reports about loci that can increase KNPS without decreasing TKW.

In this study, a novel major and stable QTL for KNPS was mapped on the wheat 2D chromosome; namely, *QKnps.sau-2D.1*. The genetic effect of *QKnps.sau-2D.1* was further confirmed in the mapping recombinant inbred line (RIL) population and in a validation population using closely linked Kompetitive Allele-Specific Polymerase chain reaction (KASP) markers. Moreover, this QTL showed a positive effect both in KNPS and TKW, resulting in a significant increase in grain yield (GY). This major and stable QTL will be useful in further cloning and in high-yield wheat breeding programs.

## 2. Results

### 2.1. Phenotypic Analyses

The statistical analysis of the phenotypic data of the KNPS in the RIL population and in the two parent lines is listed in Table 1. The KNPS of Chuannong18 (CN18) in the three years ranged from 34.93 to 58.38, with a mean value of 52.54. The KNPS of T1208 in the three years ranged from 46.60 to 74.00, with a mean value of 62.55 (Table 1). The KNPS of T1208 was higher than the KNPS of CN18 in all three years of experiments. In the RIL population, the KNPS ranged from 24.72 to 97.00, with mean values of 40.60, 64.33, and 61.02 in the three-year experiments. The broad-sense heritability (h^2^) of KNPS was 0.68, and a significant positive correlation (*p* < 0.001) was observed between the KNPS in all three years (the correlation between 2016 and 2019 was 0.3534 ***, the correlation between 2016 and 2021 was 0.3602 ***, and the correlation between 2019 and 2021 was 0.6217 ***; *** represents a significant correlation at the 0.001 probability level). The phenotypic correlations among different traits are listed in Table 2. Grain width (GW), grain length (GL), and TKW were nonsignificant correlations with KNPS in this study. Significant negative correlations were detected between KNPS and spike number per square meter (SN) (*p* < 0.01), and significant positive correlations were detected between KNPS and grain yield (GY), plant height (PH) (*p* < 0.01), and grain diameter ratio (GDR) (*p* < 0.05).

### 2.2. QTL Analysis

**Figure 1:**
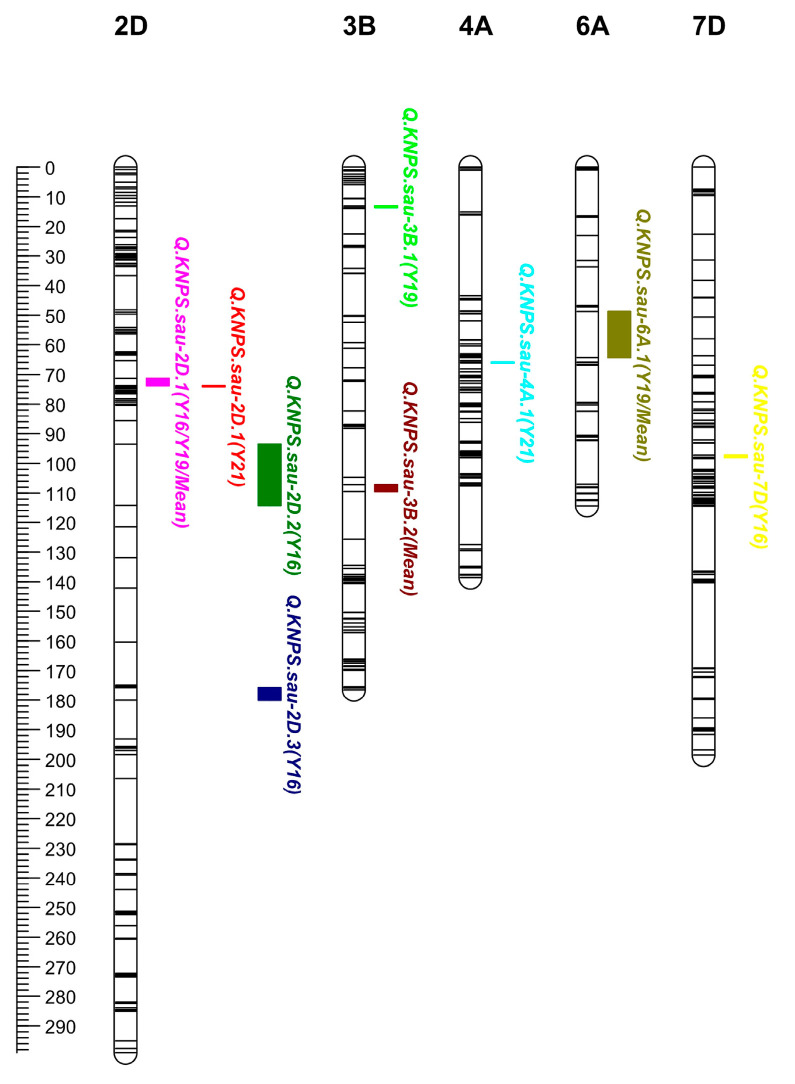
Additive QTLs for kernel number per spike (KNPS) in the genetic map. Eight additive QTLs for KNPS were mapped to chromosomes 2D, 3B, 4A, 6A, and 7D. The major and stable QTL *QKnsp.sau-2D.1* was mapped to chromosome 2D. The positions, intervals, and markers closest to these QTLs are listed in Table 3. The scale of the linkage map is 1 cM and is shown on the left.

Using the inclusive composite interval mapping-biparental populations (ICIM-BIP) method, a total of eight QTLs for the KNPS were detected, and these QTLs were mapped to chromosomes 2D (three QTLs), 3B (two QTLs), 4A, 6A, and 7D (Table 3, Figure 1). The LOD of QTLs ranged from 2.95 to 13.21, and the PVE of QTLs ranged from 2.87% to 18.10%. *QKnps.sau-2D.1* was mapped to the interval of chromosome 2D in all three years and in the mean value, explained 11.23%, 11.95%, and 12.13% of the phenotypic variation in the three years, and explained 18.10% of the phenotypic variation in the mean value (Table 3). The results suggested that *QKnps.sau-2D.1* is a major and stable QTL for KNPS. *QKnps.sau-2D.1* was initially located at 81.30–95.89 Mb (physical location: 81297795–95895626 bp on 2D) when it is compared with the Chinese Spring genome reference sequences. In addition, *QKnps.sau-2D.2* (90.5–101.5 cM) and *QKnps.sau-2D.3* (178.5–183.5 cM) were also mapped on the 2D chromosome and explained 12.80% and 12.94% of the phenotypic variation in the one-year experiment. The results suggested that these two QTLs were major QTLs but not stable for KNPS. 

Then, 27 cQTLs (combined QTLs) of KNPS were mapped using the ICIM-MET (multi-environment trial) method (Table 4). All 8 QTLs for KNPS identified by the BIP method can also be identified by the MET method (Table 3 and Table 4). Among these cQTLs for KNPS, PVE (A) ranged from 0.27% to 15.16%, while PVE (A by E) ranged from 0.01% to 1.48% (Table 4). QTL *cQKnps.sau-2D.1* (72.5–73.5 cM on 2D) corresponded to the major and stable QTL *QKnps.sau-2D.1*. The PVE (A by E) of *cQKnps.sau-2D.1* was 0.80%, while the PVE (A) of *cQKnps.sau-2D.1* was 15.16% (Table 4). It was indicated that *cQKnps.sau-2D.1* was a major and stable QTL for KNPS. 

**Figure 2:**
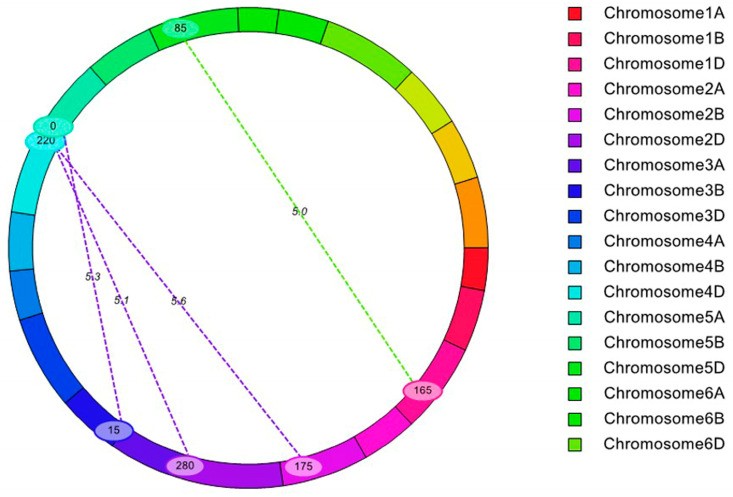
Detection of epistatic QTLs for KNPS.

Furthermore, 4 eQTLs (epistatic QTLs) for KNPS were mapped using the ICIM-EPI (epistasis) method only in the 2019 experiment and the mean value (Table S1 and Figure 2). Among these eQTLs for KNPS, the LOD ranged from 5.08 to 5.65, and the PVE ranged from 5.90% to 8.73%. However, none of the major QTLs or cQTLs mapped in this study were detected in the epistatic analyses, and the results also showed that epistasis had little effect on the KNPS.

### 2.3. KASP Marker Development and Evaluation of the Genetic Effect of the QTL

Based on the initial location of *QKnsp.sau-2D.1* and the sequences of 660K SNP markers which were different from CN18 and T1208, an SNP marker *AX-111462389* (physical location: 81.575610 Mb on 2D) was selected to develop the KASP marker. The sequence of *KASP-AX-111462389* was listed in Table S2. This KASP marker could identify the alleles in the CT population into two groups, except 48 lines were deletion: 148 lines had the “*aa*” genotype, while 175 lines had the “*AA*” genotype. The genotype “*aa*” represents *QKnps.sau-2D.1* carrying homozygous alleles from CN18, and the genotype “*AA*” represents *QKnps.sau-2D.1* carrying homozygous alleles from T1208. Then, *KASP-AX-111462389* was used to integrate into the genetic map. The integrated genetic map with the *KASP-AX-111462389* was further used to re-map the targeted major and stable QTL *QKnps.sau-2D.1*. The re-mapped results showed that the interval of *QKnps.sau-2D.1* shortened in a 1 cM genetic distance by the integrated genetic map, whose physical interval was 0.89 Mb on chromosome 2D flanked between the markers *AX-109283238* (physical location: 82.19Mb on 2D) and *AX-111606890* (physical location: 81.30 Mb on 2D) containing the newly developed KASP marker *KASP-AX-111462389*, with the PVE of 16.13%, 10.24%, 11.77%, and 15.90% on three years’ experiments and the mean values, respectively (Table S3). 

**Figure 3:**
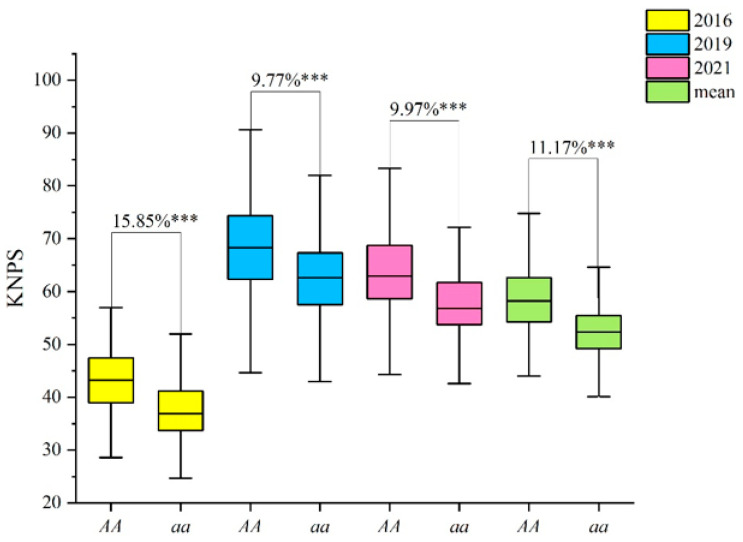
Effects of *QKnps.sau-2D.1* confirmed by *KASP-AX-111462389* in the CT RIL population. “*AA*” indicates lines which had the same genotype “*AA*” as T1208; “*aa*” indicates lines which had the same genotype “*aa*” as CN18. *** indicates significance at the *p* < 0.001 level.

With the genotype of the CT population, we evaluated the genetic effect of *QKnps.sau-2D.1*. The results showed that the phenotypic values of KNPS for lines of the *AA* genotype were 43.29, 68.64, 63.56, and 58.41 in different years and in the mean values, respectively (Table S4). However, the phenotypic values of KNPS for lines of the *aa* genotype were 37.36, 62.53, 57.80, and 52.54 in different years and in the mean values, respectively (Table S4). The results of the t-test showed that the *AA* genotypes had significantly higher phenotypic values of KNPS in all three years (15.85%, 9.77%, and 9.97%) and in the mean value (11.17%) than the aa genotypes (*p* < 0.001) (Figure 3, Table S4).

**Figure 4:**
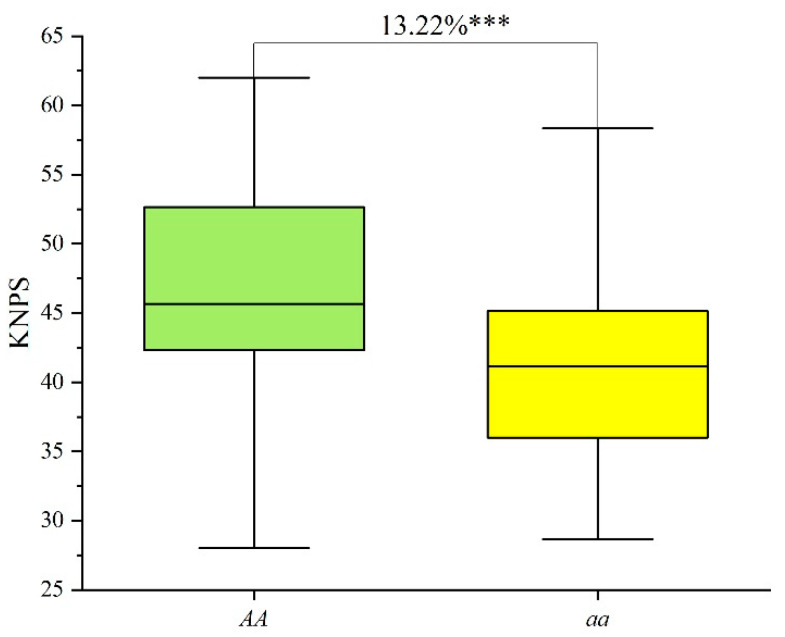
Effects of *QKnps.sau-2D.1* confirmed by *KASP-AX-111462389* in the CC RIL population. “*AA*” indicates lines which had the same genotype “*AA*” as T1208; “*aa*” indicates lines which had the same genotype “*aa*” as CN18. *** indicates significance at the *p* < 0.001 level.

The KASP marker *KASP-AX-111462389* was also used to validate the genetic effects of *QKnps.sau-2D.1* for KNPS in 102 lines of the CC RIL population. The lines with contrasting alleles at *QKnps.sau-2D.1* were also successfully classified into two groups by *KASP-AX-111462389*. Forty-two lines were *AA* genotypes, while sixty lines were *aa* genotypes (Table S5). The results showed that the phenotypic values of KNPS for lines of the *AA* genotype were 46.93, and the phenotypic values of KNPS for lines of the *aa* genotype were 41.45 (Table S5). The results of the t-test showed that the *AA* genotypes also showed significantly higher KNPS (13.22%) than the “*aa*” genotypes (Figure 4, Table S5). Overall, *QKnps.sau-2D.1* have a positive effect on KNPS.

### 2.4. Genetic Analysis of QTL QKnps.sau-2D.1

**Figure 5:**
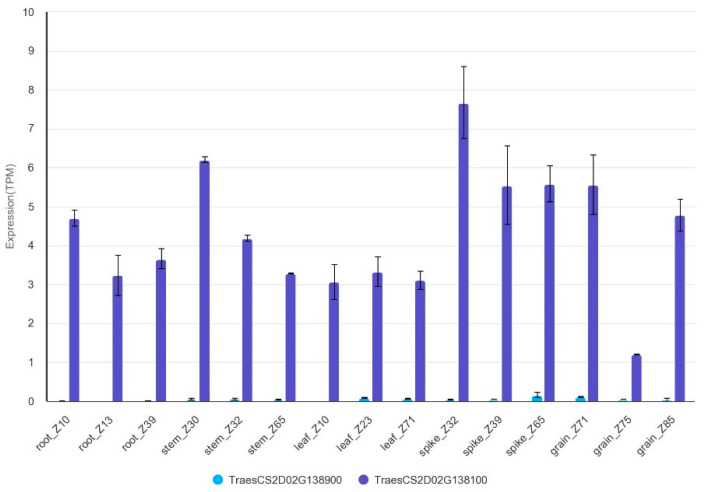
The expression of *TraesCS2D02G138900* and *TraesCS2D02G138100* in wheat.

The flanking molecular markers of *QKnps.sau-2D.1* (*AX-109283238* and *AX-111606890*) were compared with the Chinese Spring genome reference sequences. These two markers were located at 81.30–82.19 Mb of the Chinese Spring genome reference sequences (81297795, 82188769). We use them as QTL borders for identifying candidate genes. A total of 15 high-confidence genes were included in the QTL *QKnps.sau-2D.1* (Table S6). Several of these genes have been reported to be associated with grain growth and development, and several might be related to the KNPS trait. Brassinolides (BRs) affect the development of reproductive organs, and several studies have indicated that BRs have a positive regulatory effect on the yield of rice [26]. *OsBLE2*, a homologous gene of *TraesCS2D02G138900*, is involved in BR-regulated growth and development in rice [27]. *HUA2* is a homologous gene of *TraesCS2D02G138100* in *Arabidopsis thaliana* and is a key regulator of flowering time and plant reproductive organ development [28]. Moreover, *TraesCS2D02G138100* and *TraesCS2D02G138900* are expressed at all stages of wheat development, with the highest expression in the stage of spike development, but the expression level of *TraesCS2D02G138100* was much higher than that of *TraesCS2D02G138900*. (The expression data were downloaded from WheatOmics: http://wheatomics.sdau.edu.cn/, accessed on 31 August 2023). (Figure 5). It was reminded that *TraesCS2D02G138100* was most likely to be the candidate gene of *QKnps.sau-2D.1*. However, the target genes need to be verified and confirmed by further studies.

### 2.5. Effects of the QTL QKnps.sau-2D.1 Related to Other Yield Traits

Based on the genotyping results from the KASP markers, the t-test was used to analyze the effects of *QKnps.sau-2D.1* on other yield-related traits in the CT RIL population. The results showed that the *AA* genotypes had significant positive effects on TKW, GL, GW, GDR, GY, and PH compared with the *aa* genotypes, and had significant negative effects on SN (Table 5).

## 3. Discussion

### 3.1. QTL Mapping of KNPS

As one of the most important yield-related traits of wheat, several studies on QTLs related to KNPS in wheat have been reported [16,19,29,30,31]. For example, Heidari et al. [30] constructed a genetic map with 371 RAPD, SSR, RFLP, and AFLP markers, and two QTLs related to KNPS on 4A and 5B were detected. A set of 248 lines was used to construct a genetic map by using the Wheat55K SNP Assay by Xu et al. [16], and two QTLs related to KNPS were mapped and validated on chromosome 7D. These two QTLs can explain 3.82% and 6.11% of the variation in KNPS. Kuang et al. [17] mapped a QTL related to KNPS on the 4A chromosome in multiple environments in an F_2_ population derived from a cross between wheat lines 10A and BE89, and this QTL can explain 25% of the variation in KNPS; however, this QTL was not validated in a diverse panel. In this study, a genetic linkage map was constructed in a set of 371 RILs by using the Wheat55K SNP Array to perform QTL analysis for KNPS. A total of 8 and 27 QTLs related to KNPS were mapped by the ICIM-BIP and ICIM-MET methods, respectively (Table 3 and Table 4). Among these QTLs, a major and stable QTL, *QKnps.sau-2D.1*, was initially mapped on the 2D chromosome (physical location: 81.30–95.89 Mb) and can explain 18.1% of the variation in KNPS on average in multiple environments (Table 3). In order to further shorten the mapping interval, the parent lines of the CT population were genotyped by the Wheat 660K SNP Array, and an SNP marker *AX-111462389* was selected and developed into a KASP marker. The re-mapped results showed that this KNPS-related QTL was a major and stable QTL and located in a 1 cM interval between the flanking markers *AX-109283238* and *AX-111606890* (Table S4), and the physical location of *QKnps.sau-2D.1* was a 0.89 Mb interval in the region from 81.30 to 82.19 Mb on chromosome 2DS. Furthermore, The QTL-associated SNP markers of *QKnps.sau-2D.1* were analyzed, and the KASP marker *KASP-AX-111462389* showed that the *AA* genotype had a significant increase in KNPS both in the mapping population and in the validated population (Table S3, Figure 3 and Figure 4). *KASP-AX-111462389* is valuable for marker-assisted selection breeding in the future.

### 3.2. The Genetic Effects of QKnps.sau-2D.1

The most important factors of wheat yield include KNPS, TKW, and SN, and the premise of further improving wheat yield is to optimize and coordinate the relationship between these three factors [8,9]. However, recent studies have indicated that the average grain weight of wheat tends to be reduced when the kernel number is increased due to either competition or to a consistent increase in the relative proportion of grains of smaller weight potential [32]. Therefore, it will be difficult to continue to increase wheat yields substantially by simply increasing grain weight [33,34,35]. For example, Wiersma [36] found that the decrease in KNPS resulted in an increase in kernel weight; however, the 32% increase in kernel weight was completely offset by the decrease in KNPS. Moreover, negative correlations between kernel weight and KNPS have been widely reported in grain varieties other than wheat [33,37]. Previous studies have shown that there is a mutual constraint between KNPS and kernel weight, and this relationship is difficult to break, making it difficult for a single factor to play a more positive role in yield; that is, a single increase in kernel weight or KNPS makes it difficult to further increase the yield [9]. For example, Wang et al. [19] found a QTL with a negative correlation coefficient between KNPS and TKW on chromosome 4A of wheat, indicating that the increase in KNPS may lead to a decrease in TKW. Zhai et al. [4] found a QTL on chromosome 6A related to TKW and KNPS, and the genetic effect evaluation showed that the KNPS decreased by 3.05%, while the TKW increased by 8.33%. Lin et al. [20] found a QTL related to KNPS on 2DS that can increase the number of spikelets per spike by 11.38% on average. However, the study by Lin et al. [20] also found a significant negative correlation between this QTL and TKW. Therefore, only increasing KNPS without reducing TKW will lead to a significant increase in yield, but it is very rare in nature [5]. In this study, a major and stable QTL, *QKnps.sau-2D.1*, for KNPS was mapped. The lines with this QTL can significantly increase the value of KNPS (Tables S4 and S5). Moreover, in contrast to other previously reported QTLs related to KNPS [4,19,20], *QKnps.sau-2D.1* did not have significant negative effects on kernel-related traits but had significant positive effects on TKW, GL, and GW (Table 5). Moreover, t-test results showed that the genotype “*AA*” had a significant increase in GY compared with the “*aa*” genotype. The result indicated that although *QKnps.sau-2D.1* showed a significant negative effect on SN, *QKnps.sau-2D.1* showed significant effects both on TKW and KNPS. The significant increase of TKW and KNPS overcomes the yield loss caused by the decrease of SN (Table 5). 

### 3.3. The Breeding Value of QKnps.sau-2D.1

Selection is one of the most important means of breeding. In the traditional breeding process, selection is usually based on phenotype rather than genotype, because the breeders cannot directly know the genotype of an individual and can only infer it from the phenotype. The traditional selection methods were effective for some quality traits, such as disease resistance, but inefficient for quantitative traits, which often lack a clear correspondence between phenotype and genotype. Moreover, in the process of ontogeny, each trait has its specific period of expression, and many important traits can only be expressed at the later stage of development, so traditional breeding selection can only be carried out at this time. Molecular markers provide the possibility for the direct selection of genotypes [38]. If the target genes/QTLs and molecular markers were closely linked, then through the detection of the molecular marker, the genotypes of the test materials could be known [38,39,40]. This process is also known as molecular marker-assisted selection. In this study, it was found that *QKnps.sau-2D.1* could effectively increase the KNPS and thus increase the yield. By crossing the wheat lines which were carrying *QKnps.sau-2D.1* with other wheat lines with other good agronomic traits, better germplasms could be selected in the offspring However, the phenotype of KNPS cannot be confirmed until after the filling stage. In order to quickly and early confirm the character of KNPS of the test materials, corresponding molecular markers for *QKnps.sau-2D.1* were developed. The development of these markers provides important materials for marker-assisted selection of the traits of KNPS. 

## 4. Materials and Methods

### 4.1. Plant Materials

The common wheat cultivars Chuannong11 (CN11), CN18, and Chuannong17 (CN17) were all released by our lab. All three wheat cultivars exhibited many good agronomic traits, such as high spike number, low plant height, and good resistance for diseases. Therefore, these three cultivars were sown in very large areas in southwestern China [41,42]. T1208 is a 1RS.1BL translocation line with high KNPS and was developed from the cross of wheat Mianyang11 × Weining rye by our laboratory [41]. T1208 was also a high-yield wheat line and had similar adaption traits, such as growing period and plant height when it was compared with CN18. T1208 was crossed to CN18 and an RIL population including 371 lines was developed. In this study, F_12:18_ of this RIL population (CT) was used as a mapping population for QTL analyses. Another RIL population, CN17 × CN11 (CC), was used as a validation population to validate the major and stable QTLs which were detected in this study. The details of the development of these two RIL populations were reported previously [41,42]. 

### 4.2. Phenotypic Evaluation and Statistical Analysis

The CT RIL population and its two parents were planted at the experimental farm in the Qionglai District, Chengdu City (North 30°25′, East 103°28′) during the wheat planted seasons of 2016–2017, 2019–2020, and 2021–2022, following standard cultivation practices in Chengdu Basin. Each spot of the field experiments was designed as a randomized block with three replicates, 2 m length with row spacing of 25 cm (40 plants per m^2^). Pesticides and fungicides were applied in the growing season of wheat to prevent insects and diseases in order to avoid the decrease of KNPS, SN, and grain yield. The CC RIL population was planted in exactly the same way as the CT RIL population in the crop season of 2020–2021. The SN was measured directly in the field. Five plants were randomly selected in one plot and the plant height was measured. Then, ten spikes were harvested from the center of each plot and threshed manually. Then, the KNPS, TKW, GL, GW, and GDR were measured by an SC-G automatic seed analysis and 1000-grain weight analyzer (Wanshen, Hangzhou, China). These data were recruited from our previous studies [43,44]. The GY was calculated by the formula = SN × KNPS × (TKW/1000). The average values of each trait of three replicates were calculated and used in the following analysis. The broad heritability (h^2^) was determined by the software IciMapping (Version 4.2, https://isbreeding.caas.cn/rj/qtllcmapping/294445.htm, accessed on 31 August 2023). Analysis of variance (ANOVA) and Pearson’s correlation coefficient were calculated using SPSS Ver. 22.0 (IBM SPSS, Armonk, NY, USA). 

### 4.3. QTL Mapping

A linkage genetic map consisting of 11,583 SNP markers was used in this study [41]. These markers were distributed in 21 linkage groups and covered a total genetic distance of 4192.62 cM with an average interval of 0.36 cM between adjacent markers [41]. Three QTL mapping modules, ICIM-BIP, ICIM-MET, and ICIM-EPI, were used to detect the QTLs for KNPS. The QTLs for KNPS were first identified using IciMapping Ver. 4.2 BIP functionality with the ICIM method on data obtained from three years according to the methods described by Meng et al. [45]. The minimal logarithm of odds (LOD) was set at 2.5. Then, the data of the KNPS of the three years were assembled for combined QTL analysis to identify the cQTLs with additive-by-environment (A by E) interaction effects in the MET module of IciMappingVer.4.2 [45]. The parameters of cQTLs analysis were set as follows: LOD = 2.5, step = 1 cM, and PIN = 0.001. The confidence interval of each QTL was determined by the LOD peak > 2.5. QTLs that explained >10% of PVE were considered major QTLs [45,46]. Furthermore, eQTLs for KNPS were identified using the EPI module of IciMappingVer.4.2 with pre-adjusted parameters: step = 1 cM, LOD = 5, stepwise regression probability < 0.0001 [45]. QTLs were designated Q, cQ, and eQ for QTLs, cQTLs, and eQTLs with Knps (kernel number per spike), along with “sau” representing Sichuan Agricultural University and chromosome information at the end. The physical position of the QTLs was confirmed by comparing the flanking molecular markers of the QTL with the Chinese Spring genome reference IWGSC (International Wheat Genome Sequencing Consortium) sequence RefSeq v1.0 on WheatOmics (http://wheatomics.sdau.edu.cn/, accessed on 31 August 2023). The information on genes or sequences of QTL regions was also downloaded on WheatOmics, and the candidate genes in the region of the major and stable QTL were analyzed by gene annotations. The expression data of the candidate genes were searched and downloaded from the Hexaploid Wheat Expression Database IWGSC Annotation v1.1 on the website WheatOmics [47,48].

### 4.4. KASP Markers Development and QTL Validation

After obtaining the preliminary QTL mapping results, we anchored the flanking markers of the major and stable QTLs to the physical map. To develop KASP markers that could be used to trace the major and stable QTLs for KNPS, we genotyped the parents (CN18 and T1208) of the CT population using the Wheat660K SNP Array, and the SNPs mapped in the interval of stable and major QTLs were selected to develop KASP markers according to Ma et al. [39,40] and Tan et al. [49]. In this study, all KASP primers were designed on PolyMarker (http://www.polymarker.info/, accessed on 31 August 2023) according to Ren et al. [34]. The integrated genetic map with the KASP markers was further used to re-map the targeted major and stable QTLs [38]. The KASP marker closely linked with the major and stable QTL *QKnps.sau-2D.1* was used to detect different alleles at this locus in different genetic backgrounds. To evaluate the genetic effect of the stable and major QTL, all lines from the CT population and 102 lines from the CC RIL population for the screened KASP markers were used to perform genotyping. Based on the KASP genotyping results, these lines were classified into two groups: lines with homozygous alleles from T1208 (designated *AA*) and lines with homozygous alleles from the alternative parents (designated *aa*). Student’s t-test (*p* < 0.05) was used to evaluate the differences in KNPS between the two groups in each population.

## Figures and Tables

**Table 1:** Phenotypic analysis of kernel number per spike.

Traits	Years	Parental Lines		Population						*h* ^2^
		T1208	CN18	Mean	Range	SD	CV (%)	Ku	Sk	
KNPS	2016	46.60	34.93	40.60	24.72–62.58	6.39	15.73	0.19	0.29	0.68
	2019	74.00	64.33	66.04	43.00–97.00	8.87	13.43	0.13	0.19	
	2021	67.05	58.38	61.02	39.20–83.30	7.86	12.88	−0.02	0.19	
	Mean	62.55	52.54	55.84	39.41–74.77	6.11	10.95	−0.15	0.17	

KNPS: kernel number per spike; Mean: mean value in three environments; SD: standard deviation; CV: coefficient of variation; Ku: kurtosis; Sk: skewness; *h*^2^: broad-sense heritability.

**Table 2:** Correlation analysis of different traits.

	KNPS	SN	PH	GY	TKW	GL	GW	GDR
KNPS	1							
SN	−0.332 **	1						
PH	0.202 **	−0.356 **	1					
GY	0.301 **	0.089	0.125 *	1				
TKW	0.101	−0.421 **	0.604 **	0.078	1			
GL	0.089	−0.307 **	0.180 **	−0.024	0.748 **	1		
GW	−0.027	−0.237 **	0.605 **	0.081	0.659 **	0.248 **	1	
GDR	0.103 *	−0.191 **	−0.126 *	−0.06	0.416 **	0.874 **	−0.252 **	1

KNPS: kernel number per spike; SN: spike number per square meter (spikes/m^2^); PH: plant height (cm); GY: grain yield per square meter (g/m^2^); TKW: 1,000-kernel weight (g); GL: grain length (mm); GW: grain width (mm); GDR: grain diameter ratio. ** Represents a significant correlation at the 0.01 probability level. * Represents a significant correlation at the 0.05 probability level.

**Table 3:** QTL analysis for kernel number per spike by the ICIM-BIP method.

QTL	Years	CI (cM)	Left Marker	Right Marker	LOD	PVE (%)	ADD
*QKnps.sau-2D.1*	2016	70.5–73.5	*AX-110544009*	*AX-109283238*	8.49	11.23	1.69
	2019	71.5–74.5	*AX-110544009*	*AX-109283238*	11.28	11.95	3.14
	2021	71.5–74.5	*AX-109283238*	*AX-111606890*	10.29	12.13	2.69
	Mean	71.5–74.5	*AX-110544009*	*AX-109283238*	13.21	18.10	2.08
*QKnps.sau-2D.2*	2016	90.5–101.5	*AX-109422526*	*AX-108762451*	8.98	12.80	1.78
*QKnps.sau-2D.3*	2016	178.5–183.5	*AX-108808143*	*AX-110996701*	10.05	12.94	1.88
*QKnps.sau-3B.1*	2019	10.5–20.5	*AX-108760591*	*AX-109814878*	2.95	2.87	1.55
*QKnps.sau-3B.2*	Mean	88.5–89.5	*AX-110392610*	*AX-111577389*	7.17	9.19	1.45
*QKnps.sau-4A*	2021	65.5–66.5	*AX-110102976*	*AX-108907490*	3.25	3.69	1.52
*QKnps.sau-6A*	2019	55.5–65.5	*AX-108999974*	*AX-111552648*	7.01	8.07	2.54
	Mean	54.5–64.5	*AX-108999974*	*AX-111552648*	5.35	7.87	1.35
*QKnps.sau-7D*	2016	86.5–87.5	*AX-110603982*	*AX-111559194*	3.97	4.96	−1.23

KNPS: kernel number per spike; CI: confidence interval; LOD: logarithm of odds ratio; PVE: phenotypic variation explained; ADD: additive effect; if it is positive, it indicates that the genetic effect of this QTL comes from parent T1208; if it is negative, it indicates that the genetic effect of this QTL comes from CN18.

**Table 4:** QTL analysis for kernel number per spike by the ICIM-MET method.

cQTL	Interval (cM)	LeftMarker	RightMarker	LOD	LOD (A)	LOD (AbyE)	PVE	PVE (A)	PVE (AbyE)	ADD
*cQKnps.sau-1D*	221.5–238.5	*AX-111070702*	*AX-111589728*	3.54	2.98	0.57	1.04	1.01	0.03	0.61
*cQKnps.sau-2A.1*	0–2.5	*AX-111650990*	*AX-108888647*	4.02	3.93	0.09	1.53	1.35	0.18	−0.69
*cQKnps.sau-2A.2*	22.5–37.5	*AX-109477459*	*AX-109277755*	2.91	2.73	0.18	1.19	0.94	0.24	−0.57
*cQKnps.sau-2B.1*	70.5–72.5	*AX-111458829*	*AX-109359046*	3.27	3.22	0.06	1.28	1.11	0.17	−0.64
*cQKnps.sau-2B.2*	191.5–197.5	*AX-110965907*	*AX-111729522*	3.71	1.33	2.37	1.45	0.46	0.99	−0.40
*cQKnps.sau-2D.1*	72.5–73.5	*AX-110544009*	*AX-109283238*	43.03	40.57	2.46	15.96	15.16	0.80	2.33
*cQKnps.sau-2D.2*	91.5–98.5	*AX-109422526*	*AX-108762451*	9.61	3.81	5.80	2.30	1.32	0.98	0.68
*cQKnps.sau-2D.3*	178.5–182.5	*AX-108808143*	*AX-110996701*	10.17	2.47	7.70	2.34	0.86	1.48	0.57
*cQKnps.sau-2D.4*	213.5–228.5	*AX-110069106*	*AX-110090611*	2.53	2.41	0.12	1.11	0.81	0.30	−0.56
*cQKnps.sau-2D.5*	290.5–297.5	*AX-111045643*	*AX-86176576*	3.64	1.76	1.88	1.53	0.60	0.92	−0.46
*cQKnps.sau-3A*	75.5–78.5	*AX-109892124*	*AX-95003297*	2.81	2.00	0.82	1.09	0.70	0.39	−0.50
*cQKnps.sau-3B.1*	11.5–20.5	*AX-108760591*	*AX-109814878*	3.97	3.30	0.66	2.02	1.15	0.87	0.65
*cQKnps.sau-3B.2*	88.5–89.5	*AX-110392610*	*AX-111577389*	10.20	6.65	3.55	2.71	2.31	0.39	0.91
*cQKnps.sau-3D*	195.5–210.5	*AX-110503031*	*AX-109905770*	2.79	1.13	1.67	1.01	0.39	0.62	−0.38
*cQKnps.sau-4A.1*	65.5–68.5	*AX-110102976*	*AX-108907490*	4.85	3.58	1.27	1.86	1.24	0.62	0.68
*cQKnps.sau-4A.2*	75.5–78.5	*AX-110568092*	*AX-109335789*	3.54	2.21	1.33	1.22	0.76	0.46	0.52
*cQKnps.sau-4B*	14.5–17.5	*AX-111618183*	*AX-111159798*	3.50	3.27	0.23	1.43	1.13	0.31	−0.62
*cQKnps.sau-5A*	20.5–21.5	*AX-109885906*	*AX-109459767*	4.09	3.51	0.58	1.38	1.21	0.17	0.65
*cQKnps.sau-5B*	7.5–14.5	*AX-111567276*	*AX-109941289*	3.49	3.30	0.19	1.21	1.13	0.09	−0.62
*cQKnps.sau-6A*	58.5–64.5	*AX-108999974*	*AX-111552648*	14.20	12.24	1.96	5.70	4.27	1.43	1.21
*cQKnps.sau-6B*	23.5–27.5	*AX-108739547*	*AX-108925480*	2.51	2.11	0.40	0.76	0.73	0.03	0.51
*cQKnps.sau-7A*	31.5–37.5	*AX-108820421*	*AX-109916202*	2.78	2.32	0.46	0.93	0.80	0.13	0.60
*cQKnps.sau-7B*	134.5–139.5	*AX-110460794*	*AX-109908023*	4.58	4.00	0.58	1.39	1.38	0.01	0.72
*cQKnps.sau-7D.1*	86.5–87.5	*AX-110603982*	*AX-111559194*	4.85	0.79	4.07	1.30	0.27	1.03	−0.34
*cQKnps.sau-7D.2*	114.5–120.5	*AX-109728672*	*AX-110359924*	2.51	1.81	0.70	0.75	0.63	0.12	0.47
*cQKnps.sau-7D.3*	157.5–162.5	*AX-109920134*	*AX-94514350*	5.54	4.91	0.63	1.73	1.70	0.03	0.79
*cQKnps.sau-7D.4*	173.5–190.5	*AX-111523243*	*AX-108872235*	3.00	2.18	0.82	0.80	0.75	0.05	0.52

QTL represents QTL identified by the combined QTL analysis; LOD (A) represents the LOD value of additive and dominant effects of the QTL; LOD (AbyE) represents the LOD value of the interaction effect between the QTL and environment; PVE (A) represents the phenotypic variation rate explained by the additive and dominant effects of the QTL; PVE (AbyE) represents the explained phenotypic variation rate of the QTL and environmental interaction effect; ADD: if it is positive, it indicates that the genetic effect of this QTL comes from parent T1208; if it is negative, it indicates that the genetic effect of this QTL comes from CN18.

**Table 5:** Relationship between *QKnps.sau-2D.1* and kernel-related traits in CT population.

QTL	Genotypes	TKW	GL	GW	GDR	SN	GY	PH
*QKnps.sau-2D.1*	*aa*	44.17	6.87	3.47	1.98	304.01	645.71	78.70
	*AA*	48.35 ***	7.27 ***	3.51 **	2.07 ***	256.09 **	665.56 *	88.61 ***

TKW: 1000-kernel weight (g), GL: grain length (mm), GW: grain width (mm), GDR: kernel diameter ratio. SN: spike number per square meter (spikes/m^2^), GY: grain yield per square meter (g/m^2^). PH: plant height (cm). *** Represents a significant correlation at the 0.001 probability level. ** Represents a significant correlation at the 0.01 probability level. * Represents a significant correlation at the 0.05 probability level.

## Data Availability

Not applicable.

## Supplementary Materials

The following supporting information can be downloaded at: https://www.mdpi.com/article/10.3390/ijms241814289/s1.
