# Supplementary material for: Identification and Validation of a Stable Major-Effect Quantitative Trait Locus for Kernel Number per Spike on Chromosome 2D in Wheat (Triticum aestivum L.)
Source: Int J Mol Sci. 2023 Sep 19;24(18):14289. doi: 10.3390/ijms241814289 (PMC10531874; doi:10.3390/ijms241814289)
Supplement: Supplementary file 1 [file ijms-24-14289-s001.zip › ijms-2604212-supplementary.pdf]

Table S1 The results of eQTLs

Year	eQTL1	Position1 (cM)	eQTL2	Position2 (cM)	LOD	PVE (%)	ADDby ADD
2019	*eQKnps.sau-2D*	280	*eQKnps.sau-4D.1*	215	5.08	6.33	-2.83
2019	*eQKnps.sau-2B*	175	*eQKnps.sau-4D.2*	220	5.65	8.73	-3.27
2019	*eQKnps.sau-3B*	15	*eQKnps.sau-5A*	0	5.29	5.90	-2.10
Mean	*eQKnps.sau-1D*	165	*eQKnps.sau-5D*	85	5.02	8.04	1.40

Table S2 Kompetitive allele-specific PCR (KASP) markers *for QKnps.sau-2D.1*

Marker	Sequences (5' to 3')
KASP-AX-111462389-FAM	**GAAGGTGACCAAGTTCATGCT**ACCACAAGTGTTTTCGTTTTGT
KASP-AX-111462389-HEX	**GAAGGTCGGAGTCAACGGATT**ACCACAAGTGTTTTCGTTTTGG
KASP-AX-111462389-R	GACAAAGCACGAAAACTGCC

Note: The FAM probe sequence of the forward primer was GAAGGTGACCAAGTTCATGCT, and the HEX probe sequence of the reverse primer was GAAGGTCGGAGTCAACGGATT

Table S3 The re-mapping results of *QKnps.sau-2D.1*

QTL	Environment	Interval (cM)	Left Marker	Right Marker	LOD	PVE (%)	Add
QKnps.sau-2D.1	2016	76.5-77.5	*AX-109283238*	*AX-111606890*	15.19	16.13	2.21
	2019	76.5-77.5	*AX-109283238*	*AX-111606890*	7.99	10.24	2.54
	2021	76.5-77.5	*AX-109283238*	*AX-111606890*	9.87	11.77	2.63
	Mean	76.5-77.5	*AX-109283238*	*AX-111606890*	12.72	15.90	2.01

Table S4 The results of phenotyping and genotyping by *KASP-AX-111462389* in the CN18 x T1208 RIL population

QTL	Environment	Genotype *aa*	Genotype *AA*	Difference	*P* value
QKnps.sau-2D.1	2016	37.36(n=148)	43.29(n=175)	5.92	$P<0.001$
	2019	62.53(n=148)	68.64(n=175)	6.11	$P<0.001$
	2021	57.80(n=148)	63.56(n=175)	5.76	$P<0.001$
	mean	52.54(n=148)	58.41(n=175)	5.87	$P<0.001$

Table S5 The results of phenotyping and genotyping by *KASP-AX-111462389* in the CN17 x CN11 RIL population

QTL	Genotype *aa*	Genotype *AA*	Difference	*P* value
QKnps.sau-2D.1	41.45 (n=60)	46.93(n=42)	5.48	*P*＜0.05

Table S6 The 15 genes in the interval of the QTL

Gene ID name	Annotation
TraesCS2D02G138100	Hepatoma-derived growth factor-related protein 2
TraesCS2D02G138200	Transmembrane protein 115
TraesCS2D02G138300	BTB-POZ and MATH domain protein
TraesCS2D02G138400	Terpene synthase
TraesCS2D02G138500	Terpene synthase
TraesCS2D02G138600	Terpene synthase
TraesCS2D02G138700	carboxyl-terminal peptidase, putative (DUF239)
TraesCS2D02G138800	Lipase
TraesCS2D02G138900	ARM repeat superfamily protein
TraesCS2D02G139000	cDNA, clone: J075123K08, full insert sequence
TraesCS2D02G139100	Terpene synthase
TraesCS2D02G139200	Cysteine desulfurase
TraesCS2D02G139300	Microtubule-actin cross-linking factor 1
TraesCS2D02G139400	Chaperone protein dnaJ-related
TraesCS2D02G139500	Chaperone protein dnaJ-related
